# ADAM10 is expressed in human podocytes and found in urinary vesicles of patients with glomerular kidney diseases

**DOI:** 10.1186/1423-0127-17-3

**Published:** 2010-01-13

**Authors:** Paul Gutwein, Anja Schramme, Mohamed Sadek Abdel-Bakky, Kai Doberstein, Ingeborg A Hauser, Andreas Ludwig, Peter Altevogt, Stefan Gauer, Anja Hillmann, Thomas Weide, Christine Jespersen, Wolfgang Eberhardt, Josef Pfeilschifter

**Affiliations:** 1 Pharmazentrum frankfurt/ZAFES, University Hospital Goethe University Frankfurt, Frankfurt am Main, Germany; 2 Institute of Reconstructive Neurobiology, Life & Brain Center, University of Bonn and Hertie Foundation, Bonn, Germany; 3 Medical Clinic III, Nephrology, University Hospital Goethe University Frankfurt, Frankfurt am Main, Germany; 4 Institute for Molecular Cardiovascular Research, University Hospital Aachen, Germany; 5 Tumor Immunology Program, D010, German Cancer Research Center, Heidelberg, Germany; 6 Dept. of Internal Medicine, Albert-Schweitzer-Str. 33, D-48149 Münster, Germany

## Abstract

**Background:**

The importance of the Notch signaling in the development of glomerular diseases has been recently described. Therefore we analyzed in podocytes the expression and activity of ADAM10, one important component of the Notch signaling complex.

**Methods:**

By Western blot, immunofluorescence and immunohistochemistry analysis we characterized the expression of ADAM10 in human podocytes, human urine and human renal tissue.

**Results:**

We present evidence, that differentiated human podocytes possessed increased amounts of mature ADAM10 and released elevated levels of L1 adhesion molecule, one well known substrate of ADAM10. By using specific siRNA and metalloproteinase inhibitors we demonstrate that ADAM10 is involved in the cleavage of L1 in human podocytes. Injury of podocytes enhanced the ADAM10 mediated cleavage of L1. In addition, we detected ADAM10 in urinary podocytes from patients with kidney diseases and in tissue sections of normal human kidney. Finally, we found elevated levels of ADAM10 in urinary vesicles of patients with glomerular kidney diseases.

**Conclusions:**

The activity of ADAM10 in human podocytes may play an important role in the development of glomerular kidney diseases.

## Background

The important role of podocytes in the development of many glomerular diseases are documented in renal disorders like minimal change disease, focal segmental glomerulosclerosis and membranous nephropathy [1]. Adhesion molecules like the integrin α_3_β_1 _and dystroglycan are the major receptors studied today, which connect the podocytes to the glomerular basement membrane (GBM) [2]. During development L1 adhesion molecule is known to be regulated in the renal epithelium and is involved in kidney branching morphogenesis [3]. L1 adhesion molecule exists in a transmembrane form, but can also be processed into a soluble form about 200 kDa by a disintegrin and metalloproteinase (ADAM10) [4,5]. Furthermore, L1 adhesion molecule can be cleaved in vitro in the third fibronectin III domain by trypsin [6], plasmin [7] or the proprotein convertase PC5A [8], resulting in a 140 kDa and 80 kDa fragment. Interestingly, different patterns of proteolytic cleavage of L1 during nephrogenesis have been observed, but the significance of this cleavage remains unclear [3]. In addition, a 200 kDa soluble form of L1 adhesion molecule was found in patients with acute tubular necrosis and may represent a marker of distal nephron injury [9]. In the developing rat kidney ADAM10 was highly expressed in the late ureteric bud [10]. Recently we have characterized in detail the tubular and glomerular ADAM10 expression in the human kidney [11,12]. Interestingly, we found in renal allograft biopsies with histopathological diagnosis of acute interstitial rejection increased tubular ADAM10 expression, which was accompanied by high numbers of infiltrating T-cells [12]. It is known, that ADAM10 is involved in the cleavage of growth factors, adhesion molecules and cell surface receptors like Notch and their ligands Delta and Jagged [13]. In this context, two recent publications have highlighted the importance of the Notch signaling pathway in podocytes for the development of glomerular diseases. Waters et al reported, that ectotopic Notch activation in developing podocytes leads to glomerulosclerosis [14]. In addition, increased expression of the intracellular domain of Notch-1 was found in podocytes of patients with diabetic nephropathy and focal segmental glomerulosclerosis [15].

To characterize the expression of ADAM10 and its substrates L1 adhesion molecule in more detail, we analyzed their expression in a human podocyte cell line and in human renal tissue. We demonstrate that ADAM10 and L1 are expressed in human podocytes. In differentiated podocytes we detected increased amounts of mature ADAM10 and high levels of soluble L1. In addition, injuring podocytes with puromycin induced ADAM10 mediated cleavage of L1. Furthermore podocytes isolated from urines of patients with glomerular kidney diseases expressed constitutively ADAM10. Isolating urinary vesicles from healthy donors and patients with inflammatory kidney diseases, revealed increased amounts of ADAM10 expression in patients with glomerular kidney diseases.

## Methods

### Chemicals

Interferon-γ (IFN-γ) was purchased from Peprotech (Frankfurt, Germany), hyperfilms and the enhanced chemiluminescence (ECL) reagents were ordered from Amersham Pharmacia Biotech Europe GMBH (Freiburg, Germany), all cell culture nutrients were from Invitrogen/Life Technologies (Karlsruhe, Germany). The ADAM10 specific inhibitor GI254023X was assayed for inhibition of recombinant human ADAM17 and ADAM10 ectodomains as described before [16].

### Cell Culture

Human condititionally immortalized podocytes (HPC) were isolated and cultivated as previously described [17]. Prior to stimulation, cells were incubated for 16 h in RPMI 1640 medium, supplemented with 0.1 mg/ml of fatty acid-free bovine serum albumine.

### Experimental subjects

We examined the urines of a group of 7 individuals composed of 5 patients with glomerular diseases (diagnosis of patients are depicted in Table 1) and 2 healthy subjects.

**Table 1:** Clinicopathological data of patients analyzed for urinary ADAM10 expression (S-crea = serum creatinin, m = male, f = female).

Patients	Diagnosis	Age	Sex	Protenuria (g/day)	S-crea
P-1	Lupus nephritis	46	f	6,314	1,32

P-2	Morbus Wegener (not active)	72	f	0,113	0,81

P-3	IgA nephritis	72	m	3,435	5,86

P-4	IgA nephritis	38	m	1,143	4,35

P-5	Lupus nephritis	38	f	3,969	2,01

### Isolation of cells from human urines

Freshly voided urine of healthy donors and patients with glomerular kidney diseases were centrifuged at room temperature at 700 g for 10 min. The supernatant was removed by careful aspiration, the cell pellet was resuspended in 10 ml podocyte medium. The cell suspension was placed into culture flasks and incubated at 37°C in 5% CO_2_.

### Antibodies

Mouse mAb (L1-11A) to the ectodomain of human L1 adhesion molecule and polyclonal L1 were provided from Prof. Dr. Altevogt (Heidelberg, Germany). Monoclonal antibody to the extracellular part of ADAM10 was from R&D Systems (Wiesbaden-Nordenstadt, Germany). Polyclonal anti-ADAM10 antibody from eBioscience (San Diego, USA) was used for Western blot and immunofluorescence staining. Polyclonal antibodies against nephrin and podocin were kindly provided from Dr. Shuyu Ren (Bern, Switzerland). Monoclonal antibodies for β1 and α3 integrin subunits were from Chemicon (Hampshire, United Kingdom, England). WT1 antibody for immunofluorescence analysis was purchased from Santa Cruz (Heidelberg, Germany).

### Preparation of supernatants for the detection of soluble molecules

These assays were described previously [4,18]. Briefly, cell monolayers in serum-free medium were exposed to 5 μg or 10 μg puromycin to induce shedding. The ADAM10 specific metalloproteinase inhibitor GI254023X was added 15 min before treatment. Cell-free supernatants were TCA precipitated, protein samples were boiled with non-reducing sodium dodecyl sulfate (SDS) sample buffer and investigated by western blot analysis.

### Western blot analysis

Cells were lysed in ice-cold lysis buffer (50 mM Tris/HCl, pH 7.4, 150 mM NaCl, 10% glycerol, 1% Triton X-100, 2 mM EDTA, 2 mM EGTA, and 1× Complete protease inhibitors, Boehringer Complete). Supernatants were TCA precipitated. The membranes were incubated overnight with primary antibodies and bound antibodies were detected by anti-rabbit or anti-mouse/horseradish peroxidase conjugates (Santa Cruz, Heidelberg, Germany) and enhanced chemiluminescence system (Amersham, Freiburg, Germany.).

### Cytofluorography

The cells were stained with saturating amounts of mAbs, either hybridoma supernatants or purified antibodies, and phycoerythrin (PE)-conjugated goat antibodies to mouse immunoglobulins. For intracellular FACS staining, cells were fixed with 1% paraformaldehyd for 15 min at RT. Cells were washed in PBS and permeabilised with 1% Triton X-100/PBS. Primary antibodies were diluted in 1%Triton X-100/PBS and added for 30 min at 4°C to the cells. After washing the cells twice with 1%Triton-X-100/PBS, fluorescence coupled secondary antibodies were added for 20 min at 4°C in the dark. After extensive washing with 1%TX-100/PBS, stained cells were analyzed by a FACScan cell analyzer (Becton & Dickinson, Heidelberg, Germany) using Cellquest software (Becton & Dickinson, Heidelberg, Germany).

### Fluorescence microscopy (cells)

Cells were grown on coverslips and fixed with 4% paraformaldehyde/PBS or with methanol and fluorescence staining was carried out as previously described [19].

### Fluorescence microscopy (tissue)

Paraffin tissue sections were deparaffinized in xylene, rehydrated through a graded ethanol series and washed in 10 mM phosphate-buffered 150 mM saline, pH 7.4. Antigen retrieval was performed by incubating the tissue sections for 20 min in 0.01 M sodium citrate buffer, pH 6.0, in a microwave oven (500 Watt). After incubation with blocking buffer (0.1% Triton X-100/PBS containing 1% BSA and 10% horse serum) for 1 h, tissue sections were incubated with the first antibodies (diluted in 1% BSA/10% horse serum/PBS/0.1% Triton X-100) as indicated. Following washing, bound antibodies were detected by Alexa 488 conjugated goat anti-mouse (Molecular Probes, Karlsruhe, Germany) or goat anti-rabbit Cy3 (Molecular Probes, Karlsruhe, Germany) secondary antibodies. Nuclei were stained with 4',6-diamidino-2-phenylindole (DAPI, Sigma, Deisenhofen, Germany) and slides were mounted in Fluoromount G (Southern Biotechm, Birmingham, USA). Evalutation was performed by fluorescence microscopy (Keyence, Neu-Isenburg, Germany).

### siRNA

For downregulation of endogenous ADAM10 expression, the following siRNA duplex (MWG Biotech AG, Ebersberg, Germany) were used: ADAM10 construct, 5'-AGA CAU UAU GAA GGA UUA UTT-3'. As a negative control an unspecific scrambled siRNA duplex (5'-AGG UAG UGU AAU CGC CUU GTT-3') was applied.

### Transfection of siRNA

Twenty-four hours before transfection 5 × 10^4 ^cells were seeded in 6-well plates. Transfection of siRNA was carried out using Oligofectamine (InVitrogen, Karlsruhe, Germany) and 10 nM siRNA duplexes (MWG Biotech AG, Ebersberg, Germany) per well. All cells were assayed 48 h after the transfection.

### Reverse transcription-PCR analysis

RNA from urinary cells was isolated using the RNA Easy Kit according to the manufacturer's protocol (Qiagen, Hilden, Germany). Equal amounts of total cellular RNA (1 μg) were reverse-transcribed with random primer by the use of M-MuLV Reverse Transcriptase (Fermentas, St. Leon-Rot, Germany). Transcribed cDNAs were used for polymerase chain reaction (PCR) with specific primers for α3 integrin subunit (5'-CAA GGA TGA CTG TGA GCG G-3' and 5'-ATA TAG AGG TTT CCT TGG TCC-3'), β1 integrin subunit (5'-GAG AAG CTC AAG CCA GAG G-3' and 5'TCT GTT CAC TTG TGC AAG GG-3') and podocin (5'-AGA GTA ATT ATA TTC CGA CTG G-3' and 5'-TCA CTG AAT CCA AGG CAA CC-3'). PCR products were amplified using Taq DNA polymerase (NatuTec, Frankfurt, Germany) and subjected to electrophoresis using 2% agarose gels followed by ethidium bromide staining.

### Isolation of the human glomeruli

The glomeruli were isolated from the human kidney tissue according to the method of Striker and Striker [20] with minor modifications. The cortical tissue was first gently minced with a razor blade and then pushed through a steel sieve of 250-μm pore size by using a spatula. The pass-through was then filtered through a 150-μm pore size sieve and, finally, the glomeruli were collected by rinsing with PBS/1%FCS from the surface of a third sieve of 100-μm pore size. The preparation was examined under a light microscope for purity; regularly nearly 100% pure glomeruli were obtained.

### Isolation of urinary vesicles

15 ml of freshly voided urine of healthy volunteers and patients with glomerular kidney diseases were used to isolate urinary vesicles with serial centrifugation steps as described previously [19].

## Results

### Surface expression of ADAM10 and L1 is reduced during differentiation of podocytes

**Figure 1:**
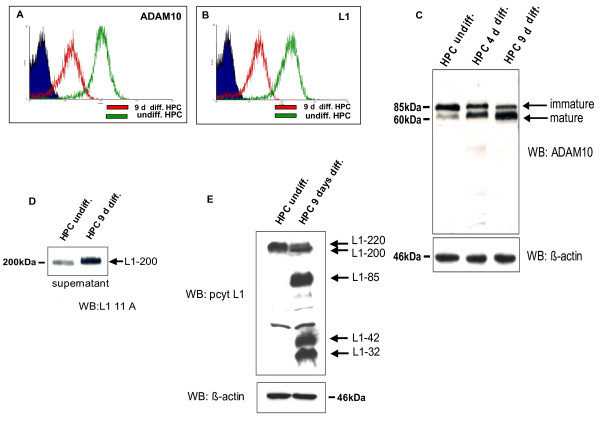
**Differentiated podocytes express decreased levels of ADAM10 and L1 adhesion molecule protein on the cell surface**. Flow cytometry histograms represents number of podocytes (cell counts, y axis) and the fluorescence intensity (x axis) of ADAM10 **(A) **and L1 adhesion molecule **(B) **and the isotype-matched control IgG antibody (filled peak) in undifferentiated (green peak) and 9 days differentiated cells (red peak). **(C)**Western Blot analysis from lysates of undifferentiated podocytes (HPC undiff.), 4 days differentiated podocytes (HPC 4 d diff.) and 9 days differentiated podocytes ((HPC 9 d diff.) with an ADAM10 specific antibody. Blots were stripped and re-probed with an antibody specific for β-actin as a loading control. **(D) **Western blot analysis of the supernatants of undifferentiated (HPC undiff.) and 9 days differentiated podocytes (HPC 9 d diff.) with L1-11A, an antibody specific for the ectodomain of L1 adhesion molecule. **(E) **Cell lysates were analyzed by western blot technique with a L1 specific antibody (pcyt). β-actin western blot was used as a loading control.

We analyzed the protein expression of ADAM10 and L1 adhesion molecule with FACS-analysis in undifferentiated and 9 days differentiated human podocytes. Interestingly, undifferentiated podocytes showed strong ADAM10 and L1 surface expression (Fig. 1A and 1B, green line). In contrast, in differentiated podocytes the surface expression of ADAM10 and L1 was significantly reduced (Fig. 1A and 1B, red line). In addition, we detected increasing amounts of mature ADAM10 in lysates of differentiated podocytes (Fig. 1C), which correlated with higher amounts of soluble L1 (Fig. 1D) and L1-32 (Fig. 1E), the cellular counterpart of soluble L1.

### ADAM10 is involved in the cleavage of L1 adhesion molecule

**Figure 2:**
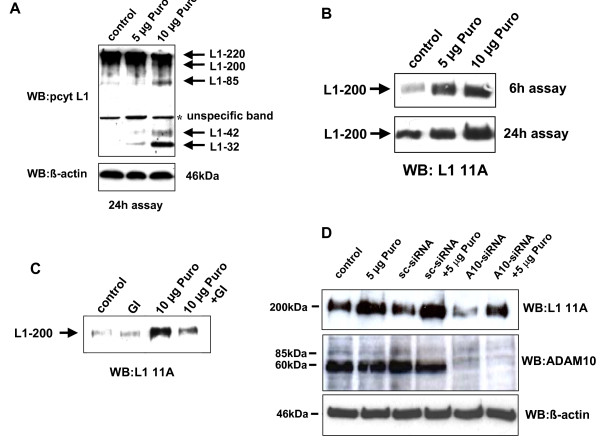
**Puromycin treated podocytes show increased levels of L1-32 and soluble L1**. **(A) **Human podocytes were treated for 24 h with 5 μg/ml and 10 μg/ml puromycin. Cells were lysed and western blot experiments were done with an antibody against the cytoplasmic tail of L1. **(B) **Human podocytes were treated for 6 h and 24 h with 5 μg/ml and 10 μg/ml puromycin (Puro), supernatants were collected and after TCA-precipitation, equal amounts of protein samples were loaded on a SDS-PAGE. Membranes were probed with L1-11A, an antibody against the ectodomain of L1. **(C) **Human podocytes were pretreated 30 min with 3 μM ADAM10 inhibitor GI254023X (GI) before incubating cells for 6 hours with 10 μg/ml puromycin (Puro). Supernatants were analyzed for soluble L1 by western blot analysis. **(D) **Western Blot analysis of soluble L1 after the transfection of ADAM10 specific siRNA in the presence or absence of 5 μg/ml puromycin (24 hour treatment). As a negative control a scrambled siRNA was used (A10 = ADAM10, sc = Scrambled, Puro = Puromycin). Efficient knockdown of ADAM10 was controlled by westernblot with ADAM10 specific antibody (A10 = ADAM10, sc = scrambled) and equal loading of the samples were determined by β-actin westernblot.

Podocyte injury occur in many glomerular diseases [21]. To injure podocytes we treated the cells with different concentrations of puromycin. Interestingly, increasing amounts of puromycin induced L1-32 in podocytes (Fig. 2A), which was accompanied by an increased amount of soluble L1 (Fig. 2B). In addition with a specific metalloproteinase inhibitor GI254023X (Fig. 2C) and ADAM10 specific siRNA (Fig. 2D) we could significantly reduce the release of L1 adhesion molecule. Interestingly, the puromycin induced cleavage of L1 was only partially inhibited by ADAM10 siRNA, whereas the constitutive release of L1 was almost completely blocked. The efficient knockdown of ADAM10 is represented in Fig. 2D.

### Urinary cells from nephrotic kidney patients express ADAM10, L1, alpha3 and nephrin

**Figure 3:**
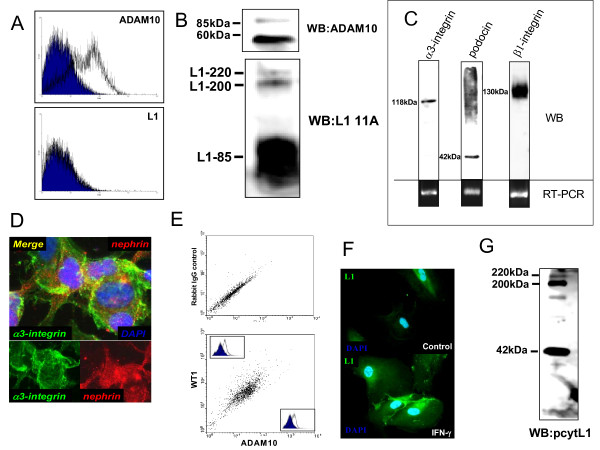
**Podocytes isolated out of the urine of patients with nephrotic syndrome express ADAM10**. Cells isolated out of the urine of a patient with nephrotic syndrome were analyzed by flow cytometry **(A+E)**, Western blot **(B+C)**, RT-PCR **(C lower panel)**, and immunofluorescence **(D+F)**. **(A) **Cells isolated from the urine were stained with ADAM10 or L1 adhesion molecule and analyzed with Cellquest software from Becton Dickinson (Heidelberg, Germany). **(B) **Urinary cells were lysed and western blots (WB) with ADAM10 and L1 (L1 11A) specific antibodies were performed. **(C) **Lower panel: RT-PCR with α3, β1, and podocin specific primers on cDNA of cells isolated from the urine. Upper panel: Western blot analysis with α3, β1 and podocin specific antibodies in lysats of cells isolated from the urine. **(D) **Immunofluorescence double staining of cells isolated from the urine with podocyte specific marker proteins α3, nephrin, podocyin antibodies. Images were documented with a Zeiss camera. **(E) **Urinary cells were investigated by intracellular FACS staining using WT1 (podocyte specific marker protein) and ADAM10 antibodies. Stained cells were analyzed with Cellquest software from Becton Dickinson (Heidelberg, Germany). **(F) **Immunofluorescence staining of untreated (control) and IFN-γ treated urinary podocytes with L1 specific primary antibodies followed by Alexa 488 coupled secondary antibodies. Nuclei of urinary podocytes were stained and visualized with DAPI. Images were documented with a Zeiss camera. **(G) **Glomeruli from human kidney were isolated and glomerular lysats were prepared, proteins were loaded on a SDS gel and western blot analysis were performed using a polyclonal antibody against the cytoplasmic tail of L1.

Viable podocytes are detectable in the urine of patients with glomerular kidney diseases [22]. Therefore we isolated urinary podocytes from patients with glomerular diseases. As demonstrated by FACS analysis (Fig. 3A) cells isolated from the urine of a patient expressed significant amounts of ADAM10 at the cell surface. Interestingly, urinary podocytes expressed mainly the mature form of ADAM10 and low levels of full-length L1 (Fig. 3B). By RT-PCR (Fig. 3C lower panel), Westernblot (Fig. 3C upper panel) and immunofluorecense (Fig. 3D) of podocyte specific marker proteins (integrin α_3_β_1 _or podocin) we confirmed that cells isolated from the urine are podocytes. In addition, by intracellular FACS staining using ADAM10 and WT1 as a specific marker for podocytes we confirmed that podocytes express ADAM10 (Fig. 3E). To determine if L1 is expressed in urinary and glomerular podocytes we performed immunofluorescence and westernblot analysis. As shown in Fig. 3F urinary podocytes only expressed low levels of L1, but L1 expression was induced after the treatment of the cells with proinflammatory cytokine IFN-γ (Fig. 3F). In addition, L1 expression was also detectable in lysates of glomeruli of normal human kidney (Fig. 3G).

### Podocytes in human renal tissue express ADAM10

**Figure 4:**
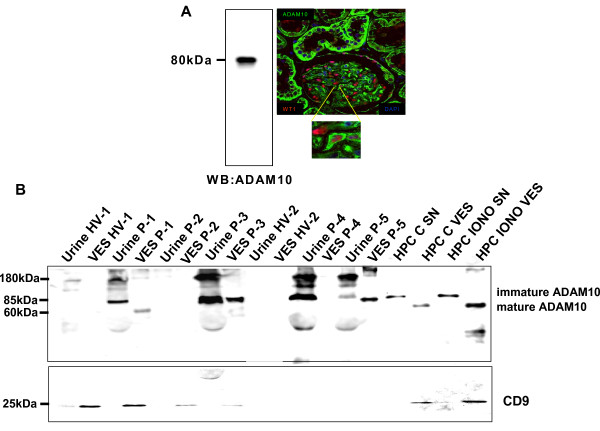
**ADAM10 is expressed in podocytes in human renal tissue**. **(A) **Glomeruli from human kidney were isolated and lysed and investigated by an ADAM10 specific westernblot (left panel). Right panel, double immunofluorescence analysis on a human kidney section with WT1 (red) and ADAM10 (green) antibodies, demonstrating ADAM10 expression in WT1 positive podocytes. **(B) Increased ADAM10 levels are found in the urine of patients with glomerular kidney diseases**. Western Blot analysis of ADAM10 expression in urine and urinary vesicles of healthy volunteers (HV 1-2) and patients with glomerular kidney diseases (number of patients P1-5, ADAM10 expression in supernatants (SN) and vesicles (VES) from untreated (HPC C) or treated with 1 μM ionomycin (HPC IONO) for 24 h. Membranes were reprobed with CD9 an specific marker protein of exosomes.

In glomeruli of human renal tissue we detected ADAM10 expression by immunohistochemistry ADAM10 expression (data not shown). To confirm, that podocytes are expressing ADAM10, double immunofluorescense analysis with a podocyte specific marker (WT1) was performed. ADAM10 expression was detectable in WT1 expressing podocytes (Fig. 4A). In addition, we isolated glomeruli out of the human kidney and investigated glomerular lysats by western blot. ADAM10 protein expression was detectable in glomeruli lysats (Fig. 4A left lane).

### ADAM10 is found in the urine and urinary vesicles of patients with glomerular kidney diseases

Exosomes in the urine are known to be a rich source for potential biomarkers [23]. Therefore we analyzed urine and urinary vesicles isolated from healthy volunteers and patients with glomerular diseases for the expression of ADAM10 and L1 adhesion molecule. We detected elevated levels of ADAM10 in urine and in urinary vesicles of patients with glomerular diseases compared to healthy volunteers (Fig. 4B). To investigate if increased amounts of ADAM10 is due to elevated levels of urinary vesicles we probed the membranes with CD9 an exosome specific marker. As shown in Fig. 4B patients with high amounts of vesicular ADAM10, demonstrated lower levels of CD9. Furthermore, we detected only in exosomes of untreated and ionomycin (induces the release of exosomes) treated human podocytes the mature form of ADAM10, whereas in the supernatants of the cells the immature form of ADAM10 could be seen (Fig. 4B). Notably, no differences in L1 expression was observed in urine and urinary vesicles of patients compared to healthy controls (data not shown).

## Discussion

In this work we demonstrated the expression of ADAM10 and L1 adhesion molecule in human podocytes. The importance of ADAM10 and L1 adhesion molecule in developmental processes are manifested in knockout models. ADAM10 knockout mice die before embryonic day 10 as a result of major defects in epithelial tissues [24]. L1 knockout mice show severe malformation of the nervous system, underlyning the importance of this molecule in the developing nervous system [25].

In the kidney it has been suggested, that L1 acts as a guidance molecule in the development of distal tubules and collecting ducts [3]. L1 knock out mice develop diverse renal malformations in addition to neurological abnormalities [26]. In contrast to previous published data [27] we detected L1 expression not only in tubular cells but also in immortalized human podocyte cell line and in primary podocytes isolated from urine of patients with glomerular disease. In the urine of patients with acute tubular necrosis (ATN) high levels of soluble L1 was detectable and the authors strongly suggest that urinary L1 could be a potential biomarker of distal injury during acute kidney injury (AKI) [9]. Beside urine and serum of patients, exosomes of body fluids may provide an avenue for the discovery of biomarkers useful for the early detection of kidney diseases and for the monitoring of treatment. We did not find significant differences in the amount of L1 in urine and urinary vesicles of healthy volunteers and patients with glomerular kidney diseases (data not shown). In contrast elevated levels of ADAM10 were detectable in urine and urinary vesicles of patients with glomerular kidney diseases. Although we have analyzed only few urine samples, this finding should be further investigated with higher numbers of urine samples from different renal diseases. Interestingly, in the urine of bladder cancer high levels of ADAM12 were detectable, suggesting ADAM12 as a promising biomarker for bladder cancer [28].

Another important substrate of ADAM10 is the Notch receptor which has also a crucial role in podocyte development. Interestingly, we found increased amounts of mature ADAM10 during differentiation of podocytes, suggesting ADAM10 as a differentiation marker for podocyte development. Importantly, a recent publication demonstrated the involvement of the Notch pathway in the development of glomerular disease [15]. In summary our finding that ADAM10 is expressed in podocytes and found in elevated levels in the urine of patients with glomerular diseases needs further investigation to clarify the involvement of this molecule in the development of glomerular kidney diseases and its usefulness as a new biomarker for glomerular injury.

## Competing interests

The authors declare that they have no competing interests.

## Authors' contributions

PG performed western blot and PCR analysis, designed and recorded the study, AS obtained the immunofluorescence (IF) data, MSA conducted the siRNA experiments, KD performed the FACS analysis, IAH collected the samples and data of the patients, AL performed double immunofluorescence staining on renal kidney sections, PA isolated urinary vesicles, SG isolated glomeruli from renal tissue, AH and TW isolated mRNA from glomeruli from human kidney, CJ and WE participated in the analysis of the study, JP coordinated and funded the study. All authors read and approved the final manuscript.
